# *AKT1* Polymorphism (rs10138227) and Risk of Colorectal Cancer in Moroccan Population: A Case Control Study

**DOI:** 10.31557/APJCP.2020.21.11.3165

**Published:** 2020-11

**Authors:** Loubna Allam, Housna Arrouchi, Fatima Ghrifi, Abdelhak El Khazraji, Ilham Kandoussi, Mohammed Amine Bendahou, Hamid El Amri, Mohammed El Absi, Azeddine Ibrahimi

**Affiliations:** 1 *Laboratoire De Biotechnologie (MedBiotech), Faculté De Medecine Et De Pharmacie De Rabat, Université Mohamed V De Rabat, Rabat, Maroc, Morocco. *; 2 *Instituts Des Analyses Génétique De La Gendarmerie Royale De Rabat, Maroc, Morocco. *; 3 *Biotechnology Laboratory (Medbiotech), Rabat Medical and Pharmacy School, Mohammed V University in Rabat, Rabat, Morroco. *; 4 *Faculté De Medecine Et De Pharmacie De Rabat, Université Mohamed V Rabat, Rabaat Maroc, Morocco. *

**Keywords:** Colorectal cancer, AKT1, LMTK3, single nucleotide polymorphisms

## Abstract

**Background:**

LMTK3 and *AKT1* each have a role in carcinogenesis and tumor progression. The analysis of single nucleotide polymorphisms of *AKT1* and LMTK3 could lead to more complete and accurate risk estimates for colorectal cancer.

**Aim:**

We evaluated the association between single nucleotide polymorphisms (SNPs) of *AKT1* and LMTK3 and the risk of colorectal cancer in a case-control study in Moroccan population.

**Methods:**

Genomic DNA from 70 colorectal cancer patients and 50 healthy control subjects was extracted from whole blood. Genotyping was performed by direct sequencing after polymerase chain reactions for the 7 SNPs (*AKT1*rs1130214G/T, *AKT1*rs10138227C/T, *AKT1*rs3730358C/T, *AKT1*rs1000559097G/A, *AKT1*rs2494737A/T, LMTK3rs8108419G/A, and LMTK3rs9989661A/G.). Study subjects provided detailed information during the collection. All P values come from bilateral tests.

**Results:**

In the logistic regression analysis, a significantly high risk of colorectal cancer was associated with TC/TT genotypes of rs10138227 with adjusted odds ratio [OR] equal to 2.82 and 95% confidence interval [CI] of 1.15 to 6.91.

**Conclusion:**

Our results suggest that the SNP *AKT1*rs10138227 could affect susceptibility to CRC, probably by modulating the transcriptional activity of *AKT1*. However, larger independent studies are needed to validate our results.

## Introduction

Colorectal cancer (CRC) is a common type of gastrointestinal malignancy. Its incidence varies from one country to another because of the diversities in their way of life, especially the diet, as well as their hereditary predisposition. The high complexity and multifactorial characteristics of colorectal cancer seriously limit its clinical management (Qingran Li et al., 2017). The risk estimate for CRC is important. The interest of its screening is significant. Indeed, it allows its prevention, or at least an effective management at a precocious stage (Jenkins et al., 2018).

One of the principal causes of the CRC is constitutive activation and deregulation of elements of the AKT/mTOR signaling pathway (*AKT1* and LMTK3). These elements are considered as major factors of tumorigenesis (Hsu et al. 2002; Page et al., 2000). They contribute to the progression of different types of cancer by promoting cell survival and proliferation. The accumulation of several molecular alterations cause tumors with specific phenotypes. These alterations give each tumor a specific phenotype that can be used as a molecular signature to reach a personalized therapy (Slaoui et al., 2014)

The main role of LMTK3 is to regulate the transcriptional activation of ESR1 by inhibiting PKC, resulting in a decrease of AKT (Ser473) responsible for the stabilization of FOXO3, which allows the increase of the Erα transcriptional activity (Giamas et al., 2011).

LMTK3 and *AKT1* each have a role in carcinogenesis and tumor progression. Deregulation of these two genes has been detected in a wide range of human malignancies, including that of the breast, lung (anaplastic small cell), bladder (Mundhenk et al., 2011), and digestive tract (Gastric, colorectal (Ying et al., 2015; Shi et al., 2014; Zhang et al., 2015; Wakatsuki et al., 2013; Shi et al. 2013)). These molecules play a critical role in the development of cancer therapy.

Understanding of upstream regulators, Downstream targets and in vivo functions of AKT promote the treatment of the diseases generated (cancer (Avan et al., 2014; De Marco et al., 2013; Kim et al., 2012), diabetes (Devaney et al., 2011), as well as schizophrenia (Norton et al., 2007; Ikeda et al., 2004), ...). It is assumed that possibly functional SNPs in AKT genes could modify its function and/or its protein expression, thus modifying the response and sensitivity to cancer. The analysis of single nucleotide polymorphisms (SNPs) of *AKT1*, an isoform of AKT, and LMTK3 is only a beginning of the molecular pathogenesis understanding and the revelation of mechanisms leading to susceptibility to cancer. For reinforce biological plausibility, the variants combination of the LMTK3/*AKT1* networks could give rise to more complete and accurate risk estimates for CRC than those can be obtained from a single variant.

Numerous epidemiological and biological studies had shown CRC risk factors. The available knowledge is still insufficient to reveal its mechanism. A candidate gene approach focused on the AKT/mTOR pathway should allow to discover low penetrance alleles that would increase susceptibility to cancer or reduce the age of the onset disease. Variations in AKT play an important role in carcinogenesis. However, no study has been conducted to determine if *AKT1* and LMTK3 variants are also associated with the CRC risk. This suggests the idea of realizing tests able to detect the SNPs effects of these genes, whose products are involved in the control of the *AKT1*/mTOR pathway and the cellular response to DNA damage. In this optics, therefore, we conducted a combination study to evaluate the effect of five *AKT1* SNPs and two LMTK3 SNP on the CRC risk in a case-control study. We have genotyped a series of subjects in the Moroccan population with sufficient data. The clinico-pathological parameters were analyzed to explore the association of these polymorphisms with the prognosis CRC and thus identify new biomarkers for the risk of CRC.

## Materials and Methods

*Ethical considerations*

The study was approved by the Rabat Institutional Review Boards (IRBs) conducted under FDA’s Bioresearch Monitoring (BIMO) Program.

*Characteristics of the studied population*

The study consisted of 70 Moroccan patients with colorectal cancer, diagnosed, and confirmed histologically. Who has not undergone any form of radiotherapy and/or preoperative chemotherapy. They were collected in the period 2017 to 2018. The 50 healthy controls used in this study were anonymous Moroccan blood donors without cancer, randomly selected from the Ibn Sina Rabat Hospital Center. The information about patients and samples were obtained with the informed consent and approval of the Ethics Committee. 

The study protocol was approved by the relevant committees for the use of human subjects in research. The samples (10 ml of blood, collected in vacutainer tubes containing EDTA), were obtained from the study subjects, a structured questionnaire was used to obtain detailed information on age, sex, disease history, size, and stage of the tumor. All participants were also measured for their current weight. The clinical and pathological features of the patients are summarized in Table 1. Histological data have been evaluated according to the criteria of the World Health Organization. The staging of postoperative pathology specimens by the tumor, node, and metastasis (TNM), was performed according to the criteria of the 7th Edition of the International Union Against Cancer (UCC) 2010 (AJCC) 2010) criteria. This study was approved by the Ethics Committee of the Faculty of Medicine and Pharmacy of Rabat.

*DNA extraction and Dosage*

Genotyping of CRC patients and healthy controls was performed by genomic sequencing for the seven selected polymorphisms of the *AKT1* and LMTK3 genes. Genomic DNA is extracted and purified from whole blood using the kit “ISOLATE II Genomic DNA Extraction” according to the manufacturer’s instructions (Bioline). The quantity and quality of DNA were measured by the Nanovue Plus spectrophotometer (Biochrom, Holliston, USA).

Between 10 to 100 ng genomic DNA were used for each polymerase chain reaction (PCR). DNA was amplified in a SimpliAmp ™ Thermal Cycler PCR System (Applied Biosystems ™ A24811). These reactions were carried out in 50 μL of MyTaq ™ Mix Reaction kit (Bioline), containing all the reagents (including stabilizers) and the specific primers for each SNP (10 μM). The reaction mixtures were heated at 94°C for 3mn, followed by 35 cycles at 94°C for the 30s, Tm °C of each SNP for 30-45s, and 72 C for 30s. In the end, the reactions were prolonged for 5 min at 72°C.

*DNA sequencing and genotyping*

PCR products were purified by ExoSAP-IT and sequenced by BigDye® Terminator (BDT) v3.1 (Cycle Sequencing Ready Reaction Kit) according to the manufacturer’s instructions (Applied Biosystems). The sequence reaction was performed in an automated thermal cycler(96°C for the 60s, followed by 25 cycles at 96°C for 10s, 50°C for 5s, and 72°C for 4min). The reaction mixtures were purified by the BigDye X Terminator purification kit (10 μl of DNA, 45 μl of the SAM ™ solution, and 10μl of XTerminator). In the end, the detection of the sequence products was carried out by capillary electrophoresis in the ABI3500 DNA analyzer (Applied Biosystems).

*Statistical analyzes*

Statistical analysis was performed using SPSS software (version 19.0, SPSS Inc., Chicago, IL, USA). Hardy-Weinberg equilibrium analyzes were tested by SnapStat software (https://www.snpstats.net/start.htm).The Hardy-Weinberg equilibrium has been assumed for a p-value greater than 0.05. Logistic regression models were used to estimate the association between genotypes (polymorphisms) and the risk of developing a CRC. To evaluate the strength of this association, Odds Ratios (ORs), and 95% confidence intervals (CIs), were calculated. Values of p <0.05 were considered statistically significant.

## Results

*Demographic characteristics*

The general characteristics of the cases and control subjects in this study are listed, in Table1. The mean age (SD) of the case and control groups was respectively 60.34 (11.269) and 57.96 (11.854).No significant difference was detected, in the age and the sex distribution between the two groups (P> 0.05).Compared to controls, the cases were more likely to have a lower weight 45 kg (P = 0.0076)). This may be due to decreased weight during the illness (weight loss being one of the symptoms of cancer). According to clinical information, the percentage of patients at clinical stages 0 and I (68.60%) was twice as higher than in clinical stages II and III (31.40%). This corroborates with the percentage of negative LNM (84.3%), which is five times higher than positive ones (15.7%). According to the same information, patients with tumor size <5 (67.1%) were twice as large as those with size> 5 (32.9%).

*Characteristics of the studied population*

A total of 70 patients with CRC and 50 control healthy matched in the population were genotyped successfully for 5 SNPs of *AKT1* (rs1130214G/T, rs10138227C/T, rs3730358 C/T, rs1000559097G/A, rs2494737A/T), and for two SNP of LMTK3(rs8108419G/A, rs9989661A/G). The Genotypes frequencies were in accordance with Hardy-Weinberg equilibrium (all P>0.05), except the genotype *AKT1*rs1000559097 of cases (P <0.05).

*SNPs association and colorectal cancer risk*

As shown in Table2, a significant difference, between individuals with CRC and healthy controls, were observed for *AKT1* rs10138227 (p = 0.021).

The risk of colorectal cancer was significantly higher in individuals with the CT-TT combined genotype of *AKT1*rs10138227 compared to the CC homozygous genotypes (Odds Ratio, OR=2.82, 95% Confidence Interval (CI) = 1.15-6.91), p=0.021). However, no variant genotypes of other SNPs (*AKT1* (rs1130214, rs3730358, rs1000559097, rs2494737), and LMTK3 (rs8108419, rs9989661)) were associated with a risk of colorectal cancer (Table 2). Data analysis showed that only rs10138227 was significantly associated with colorectal cancer risk. No evidence of rs10138227 association with sex (P=0.055), age (P=0.37), weight (0.43) and clinico-pathological parameters as tumor size(0.22), stage(0.92) TNM (depth of invasion and lymph node metastasis) (P=0.7) was not obtained (Table 3).

**Table 1:** Frequency Distributions of Selected Characteristics of Colorectal Cancer Cases and Cancer-Free Controls. CI, confidence interval; OR, odds ratio. The results were in bold if the 95% CI excluded 1 and P < 0.05

Cases/ Controls	Variables		OR (95% CI)	P-value
	Sex			
	Female	Male		
Controls	20 (44.4%)	30 (40%)	1	
Cases	25 (55.6%)	45 (60%)	1.20 (0.57-2.53)	0.63
	Age			
	<60	>60		
Controls	28 (46.7%)	22 (36.7%)	1	
Cases	32 (53.3%)	38 (63.3%)	1.51 (0.73-3.14)	0.27
	Weight			
	<45	>45		
Controls	10 (25%)	40 (50%)	1	
Cases	30 (75%)	40 (50%)	0.33 (0.14-0.77)	0.0076

**Table 2:** Logistic Regression Analysis of Associations between the Genotypes of *AKT1* and *LMTK3*, and Colorectal Cancer Risk. CI, confidence interval; OR, odds ratio. The results were in bold if the 95% CI excluded 1 and P < 0.05

Model	Genotype	Cas/Control		OR (95% CI)	P-value
		Cas	Control		
*AKT1*rs1130214G/T					
	G/G	39 (55.7%)	22 (44%)	1	0.21
	T/G-T/T	31 (44.3%)	28 (56%)	1.60 (0.77-3.33)	
*AKT1*rs10138227C/T					
	C/C	60 (85.7%)	34 (68%)	1	0.021
	T/C-T/T	10 (14.3%)	16 (32%)	2.82 (1.15-6.91)	
*AKT1*rs3730358C/T					
	C/C	41 (58.6%)	32 (64%)	1	0.55
	T/C-T/T	29 (41.4%)	18 (36%)	0.80 (0.38-1.68)	
*AKT1*rs1000559097G/A					
	G/G	39 (55.7%)	22 (44%)	1	0.21
	A/G-A/A	31 (44.3%)	28 (56%)	1.60 (0.77-3.33)	
*AKT1*rs2494737A/T					
	A/A	33 (47.1%)	24 (48%)	1	0.93
	A/T-T/T	37 (52.9%)	26 (52%)	0.97 (0.47-2.00)	
LMTK3rs8108419G/A					
	G/G	38 (54.3%)	26 (52%)	1	0.8
	A/G-AA	32 (45.7%)	24 (48%)	1.10 (0.53-2.27)	
LMTK3 rs9989661A/G					
	AA	47 (67.1%)	28 (56.0%)	1	0.215
	AG-GG	23 (32.9%)	22 (44.0%)	0.623(0.295-1.317)	

**Table 3:** Association between the Genotype *AKT1*rs10138227 and the Clinicopathological Variables. CI, confidence interval; OR, odds ratio. The results were in bold if the 95% CI excluded 1 and P < 0.05

Genotype	Variables		OR (95% CI)	P-value
	Sex			
*AKT1*rs10138227	Female	Male		
C/C	31 (68.9%)	63 (84%)	1	
T/C-T/T	14 (31.1%)	12 (16%)	0.42 (0.17-1.02)	0.055
	Tumor size			
*AKT1*rs10138227	<5	>5		
C/C	42 (89.4%)	18 (78.3%)	1	
T/C-T/T	5 (10.6%)	5 (21.7%)	2.33 (0.60-9.06)	0.22
	Tumor stage			
*AKT1*rs10138227	0-I	II-III		
C/C	41 (85.4%)	19 (86.4%)	1	
T/C-T/T	7 (14.6%)	3 (13.6%)	0.92 (0.22-3.97)	0.92
	LNM			
*AKT1*rs10138227	Negative	Positive		
C/C	51 (86.4%)	9 (81.8%)	1	
T/C-T/T	8 (13.6%)	2 (18.2%)	1.42 (0.26-7.78)	0.7
	Age			
*AKT1*rs10138227	<60	>60		
C/C	49 (81.7%)	45 (75%)	1	
T/C-T/T	11 (18.3%)	15 (25%)	1.48 (0.62-3.57)	0.37
	Weight			
*AKT1*rs10138227	<45	>45		
C/C	33 (82.5%)	61 (76.2%)	1	
T/C-T/T	7 (17.5%)	19 (23.8%)	1.47 (0.56-3.85)	0.43

## Discussion

The gene network of *AKT1*/mTOR signaling pathway plays a crucial role in cancer development. Polymorphisms within this network may affect the expression or function of the corresponding protein and thus potentially affect the risk of developing different types of cancers (Karen et al., 2006). However, the role of genetic variations in this network is not yet completely understood. This study evaluated the effect of six SNPs of *AKT1* and LMTK3 selected on the CRC risk. The main discovery was a significant association between individuals with CRC and healthy controls for *AKT1* rs10138227 (p=0.021). The heterozygous CT-TT combined genotypes of *AKT1*rs10138227 were associated with colorectal cancer risk. However, no variant genotypes of other SNPs (*AKT1* (rs1130214, rs3730358, rs1000559097 and rs2494737) and LMTK3 (rs8108419, rs9989661)) has not been associated with a risk of CRC.

Somatic aberrations of PI3K/AKT/mTOR pathway genes have been frequently observed, in various cancers. Therefore this pathway has been extensively studied as a mechanism of tumorigenesis and the target of cancer treatment.

AKT, the homolog of the murine thymoma viral oncogene v-AKT, which located in human chromosome 14q32.32, encodes a protein of 480 amino acids (Kim et al., 2012). AKT is an important effector of the PI3K/AKT/mTOR signaling pathway. A genetic mutation or an abnormal expression of this protein may alter various cellular processes that are related to cancer initiation and progression(De Marco et al., 2013). AKT SNPs would be associated with the susceptibility and/or prognosis of various types of cancer, including nasopharyngeal carcinoma(Zhang et al., 2014), oral squamous cell carcinoma (Wang et al., 2015), non-small cell lung cancer (Li et al., 2013; Kim et al., 2012), pancreatic ductal adenocarcinoma (Avan et al., 2014), and gastric cancer (Wang et al., 2014). In addition, data analysis showed that only one variant of AKT1rs10138227 gene was significantly associated with colorectal cancer risk.

No evidence of rs10138227 association was retained, with sex, age, and lymph node metastasis stage(TNM). However, in the logistic regression analysis, no statistical evidence was found to detect the multiplicative interactions between these SNPs, which may be due to the relatively small size of the effect of some selected SNPs.

Considering ethnic origin and environmental factors of (type and nature of food ...) the Moroccan population, we realized a group analysis of AKT pathway variants and the CRC risk. To our knowledge, this is the first study analyzing the association of the *AKT1* and LMTK3 genes of the PI3K/AKT/mTOR pathway with the CRC risk. We have, therefore, demonstrated the importance of the *AKT1* polymorphism, as a predictive marker of CRC risk in patients who develop the disease at an earlier stage.

Given that the PI3K/AKT/mTOR pathway plays an important role, in balancing growth and cell death. It is possible that the germline polymorphisms of these genes influence the occurrence of cancer in humans. The reason, for several studies, has been realized.

In the study by Kim et al., (2010), who evaluated the association between a set of 14 polymorphisms of PI3K/PTEN/AKT/mTOR pathway genes and CRC, no association was observed, between the selected SNPs of this pathway (including rs1130214 and rs2494737) and survival of patients with colorectal adenocarcinoma.

Similarly, in the study by Wang et al., (2016), no genotype variant from rs10138227 was associated with a risk of gastric cancer in a population in eastern China. This same SNP was not associated with any of the metabolic syndrome endophenotypes (diabetes) (Devaney et al., 2011).

The study by Folios Loupakis et al., (2012), suggested that LMTK3 could be an independent prognostic factor for patients with metastatic colon cancer and would pose the problem of possible disparities depending on the location of the primary tumor. Other studies such as that of Zhang et al., (2012), demonstrated that there is no significant association between colon cancer and LMTK3 rs9989661 in men and rs8108419 in both sexes. This confirms the result of our study. In contrast, they presented an association of LMTK3 rs9989661 in women with Colon Cancer. Design studies of the 3D structure and LMTK3 and *AKT1* inhibitors (Allam et al., 2017; Allam et al., 2018) may be useful to block the spread of cancer in patients with CRC.

When reviewing the results of this study, it is also suitable to keep in mind several potential limitations. First, since our study population consisted of a small number of patients, our first results should be studied in additional studies with larger samples.

Furthermore, the limited size of the sample may result in a decrease in the detection of lower genetic effects in carcinogenesis, and which, must be confirmed in other studies involving large populations. Second, in this study, we selected seven variants of two genes in the AKT/mTOR tumor network. These variants did not cover all functional SNPs. Thus this study might have overlooked some important variants. For this purpose, additional studies on other variants and other genes would corroborate the role of this important network in the genetic etiology of CRC. Thirdly, the case-control study may include selection biases and inherent information. Owing to insufficient diversity of medical records (clinical stages), we were unable to collect patients with advanced tumor stage. This latter could have provided more information on CRC carcinogenesis. Furthermore, other in vitro and in vivo experiments are needed to unravel the molecular mechanisms of the genetic association that we have advanced in this study.

In summary, we have shown that rs10138227 *AKT1* gene variation in the PI3K/AKT/mTOR pathway may be associated with the risk of colorectal cancer. The wild genotype of this variant increased the risk of CRC. In conclusion, our study concluded, that genetic variants can alter the expression and activity of *AKT1*, leading to susceptibility to CRC.

These results provide experimental evidence to support AKT as a potential biomarker for specific types of CRC and also to identify high-risk subgroups of patients likely to benefit from personalized prevention and treatment. Exploration of more variants of SNPs will further contribute to confirming the interest of this network and its association with CRC risk.
